# Characterization of handheld disinfectant sprayers for effective surface decontamination to mitigate severe acute respiratory coronavirus virus 2 (SARS-CoV-2) transmission

**DOI:** 10.1017/ice.2020.1423

**Published:** 2021-01-13

**Authors:** Seong Chan Kim, Dong-Bin Kwak, Thomas Kuehn, David Y.H. Pui

**Affiliations:** 1 Department of Mechanical Engineering, University of Minnesota, Minneapolis, Minnesota, United States; 2 School of Science and Engineering, The Chinese University of Hong Kong, Shenzhen, Guangdong, China

Much research has been conducted to prove the airborne transmission of severe acute respiratory coronavirus virus 2 (SARS-CoV-2)^1,2^; however, fomite transmissions, in which infected expiratory droplets that have settled on surfaces are transmitted to the next individual, are also considered one of the main sources of viral spread.^3,4^ Van Doremalen et al^5^ evaluated the persistence of SARS-CoV-2 on plastic, stainless steel, and cardboard surfaces, and the results showed that the virus survived up to 72 hours,^5^ which made surface decontamination a practical way to mitigate virus spread. Surface disinfection is officially recommended as one of the main COVID-19 preventive measures by authorized organizations such as the World Health Organization, the Centers for Disease Control and Prevention, and the European Center for Disease Control.

With concerns of cross contamination by conventional surface-wiping methods, not to mention its excessive time and labor requirement, a disinfectant spray method has become a common decontamination practice. Various types of disinfectant spray methods are used to decontaminate frequently contacted surfaces as a daily routine by janitorial staff.^6-10^ However, the effectiveness of these sprayers has not been investigated to optimize disinfectant droplet deposition on surfaces being routinely decontaminated. This report documents a method to characterize the sprayed droplet size and distribution on test surfaces and provides the optimum spray application practice to achieve the highest effectiveness of disinfectant sprayers.

## Methods

Three different types of disinfectant sprayers were tested to characterize droplet size and coverage distribution on a classroom desk (152 × 48 × 73 cm^3^). Fluorescein sodium salt (C_20_H_10_Na_2_O_5_, Cas 518-48-8, Mw = 376.27 g/mol) was dissolved in the disinfectant solution (HB quat disinfectant cleaner concentrate, 3M, St Paul, MN) with a concentration of 0.18 g/L for analysis using a fluorescence microscope (Eclipse Ti, Nikon, Tokyo, Japan). Next, 15 clean cover glasses (18 × 18 mm^2^) were deployed in 3 rows and 5 columns on the surface of the table to collect the disinfectant droplets generated by the test sprayers. The droplet size and area coverage were calculated using image processing from the fluorescence microscope images. We evaluated 3 different spraying methods: a trigger sprayer and an electrostatic sprayer (VP200ES, Victory Innovation, Eden Prairie, MN) with and without electrostatic charging. The trigger sprayer was applied 3 times from the front and once from the side of the desk while moving along the edge (10 seconds along the front and 5 seconds along the side). The electrostatic sprayer with the droplet charge on or off was continuously applied by walking along the front for 10 seconds or the side for 5 seconds. In all cases, the spray was released horizontally, and the nozzle tip location was maintained at a height of 1 m and 0.2 m away from the edge of the desk for consistent comparison. The room temperature and relative humidity were measured as 24°C and 35%.

## Results

**Fig. 1.**
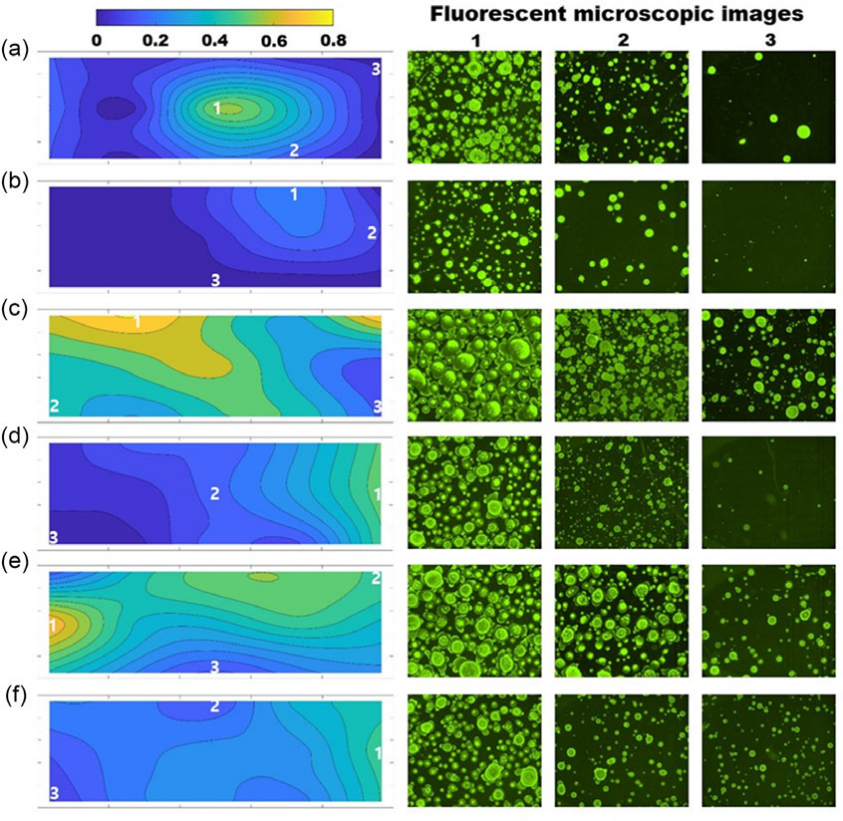
Deposition profiles and fluorescent microscopic images of disinfectant droplets collected on a classroom desk with various sprayer types and spray directions: front (from top of the figure) and side (from right of the figure). The droplet depositions were sampled at 15 locations and the microscopic images that show the highest (1), medium (2), and lowest (3) depositions from each case are shown on the right. (a) Trigger sprayer (front), (b) Trigger sprayer (side), (c) electrostatic sprayer with charge on (front), (d) Electrostatic sprayer with charge on (side), (e) electrostatic sprayer with charge off (front), and (f) Electrostatic sprayer with charge off (side). The contour color bar represents local disinfectant coverage.

Figure 1 shows the disinfectant droplet distribution profiles and fluorescence microscopic images at high, medium, and low concentration locations for each case. Each image has the same magnification representing the image size of 4.15 × 3.50 mm^2^. As shown in the deposition profiles, the trigger sprayer (Fig. 1a and b) cannot spread disinfectant droplets uniformly over the desk due to the low liquid flow rate as well as their short projection distance. The maximum area coverage was ˜60% at a limited number of locations and the droplet size was 100–300 µm. Based on this information, multiple applications at appropriate locations with trigger sprayers are recommended to perform effective surface disinfection. However, it is not practical in real situations to sanitize multiple desks with limited time and cleaning staff. The electrostatic sprayer yields much better results in terms of droplet distribution and area coverage regardless of the electrical charge, on (Fig. 1c and d) or off (Fig. 1e and f). The front spraying results show a more uniform droplet distribution with higher coverage than the side-spraying results. The front spraying still shows some nonuniformity due to spray pulsations, which can be improved by swaying the sprayer laterally while walking along the front of the desk. The side-spraying results clearly show the limited reach of the electrostatic sprayer at the opposite end of the desk, which causes insufficient coverage on the surface. To remedy this issue, side spraying needs to be conducted from both sides of the desk.

In this report, we characterized 3 types of disinfectant sprayers (a trigger sprayer and an electrostatic sprayer with droplet charging on or off) in terms of droplet size and area coverage on a classroom desk. Fluorescein was dissolved in the test disinfectant solution to be detected by a fluorescence microscope. The droplets were sampled using cover glasses distributed on the desk. These fluorescent droplets were analyzed using image processing to calculate the droplet size and area coverage at each location. The deposition profile results show more uniform deposition when sprayed from the front of the desk, while the side-spraying results show the limited reach of the sprayer. For optimum electrostatic sprayer performance, sway laterally while spraying from the front of the desk or to apply from both sides. The trigger sprayer is not suggested for the high-volume disinfection procedure due to its poor deposition performance here despite its availability and cost-effectiveness.
